# *Pediatric Neurology Briefs*: Year in Review

**DOI:** 10.15844/pedneurbriefs-33-1

**Published:** 2019-12-31

**Authors:** John J. Millichap

**Affiliations:** 1 Division of Neurology, Ann & Robert H. Lurie Children’s Hospital of Chicago, Chicago, IL; and Departments of Pediatrics and Neurology, Northwestern University Feinberg School of Medicine, Chicago, IL

**Keywords:** Neurology, Pediatrics, Child Development, Nervous System Diseases, Brain Diseases

## Abstract

In 2019, the mission of *Pediatric Neurology Briefs* (PNB) remains the same: “PNB is a continuing education service designed to expedite and facilitate the review of current scientific research and advances in child neurology and related subjects.”

In 2019, the mission of *Pediatric Neurology Briefs* (PNB) remains the same: “PNB is a continuing education service designed to expedite and facilitate the review of current scientific research and advances in child neurology and related subjects.” Last year, the publishing model of PNB evolved even further. The canonical version of the article was published online as soon as it was accepted and typeset. The published articles are collected into online volumes and an annual collection for print will be available at the end of every year.

PNB has been published since 1987 and consists of 32 volumes with greater than 3700 articles and includes over 10,000 citations referencing the works of approximately 28,000 scholarly authors. PNB was relaunched as an open access, peer-reviewed, journal with an expanded editorial board in January 2015. The Editors of PNB select source articles using criteria that include recent publication in a peer-reviewed journal and a topic of clinical value to practicing pediatric neurologists. Contributing Editors provide detailed summaries of published articles, followed by commentaries based on their experience and corroborated by appropriate supplementary citations. Also in 2015, PNB launched a new website and content management system capable of organizing peer-review and providing improved indexing, DOI assignment, and online full-text article view. Prior to the internet, the Editor periodically compiled and published the PNB articles in book form with index, according to subject heading and in chronological order [1-3].

In 2016, digitization of the 30 years of back issues was completed. There are now over 3700 full text open access articles available on the journal website. Also in 2016, PNB was selected for inclusion in PubMed Central® (PMC). PMC is a free archive of biomedical and life sciences journal literature at the U.S. National Institutes of Health's National Library of Medicine (NIH/NLM). The most highly accessed articles published in 2018 covered topics related to the microbiome [4], cannabidiol [5], anticonvulsant pharmacokinetics [6], and the use of EEG in autism diagnosis [7].

The Editors would like to take this opportunity to sincerely thank the 2018 Contributing Editors for their effort and expertise (listed below in alphabetical order):

Murtaza N. Ali

Zarraf Arefeen

Joanna Blackburn, MD

Robyn Blackford, RDN, LDN

Nada Boutrid, PhD, MD

Daniel A. Freedman, DO

Rebecca Garcia-Sosa, MD

Cristiana A. Guido, MD

Syed Mosab Kazmi

Arpita Lakhotia, MD

John J. Millichap, MD

Divakar S. Mithal, MD PhD

Stephen L. Nelson, MD, PhD

Meghan O’Neill, MD

Anup D. Patel, MD

Hakim Rahmoune, MD

Roberto Refinetti, PhD

Jennifer P. Rubin, MD

Safiullah Shareef, MD

Talia Shear, MD

Alberto Spalice, MD, PhD

Egidio Spinelli, MD

Brittani M. Wild, DNP, FNP-C

Topic experts interested in contributing to PNB as authors or reviewers are invited to contact the Editor at j-millichap@northwestern.edu.

## Disclosures

The author has declared that no competing interests exist.
